# Dose-dense adjuvant chemotherapy in early breast cancer patients: 15-year results of the Phase 3 Mammella InterGruppo (MIG)-1 study

**DOI:** 10.1038/s41416-020-0816-8

**Published:** 2020-03-31

**Authors:** Eva Blondeaux, Matteo Lambertini, Andrea Michelotti, Benedetta Conte, Marco Benasso, Chiara Dellepiane, Claudia Bighin, Simona Pastorino, Alessia Levaggi, Alessia D’ Alonzo, Francesca Poggio, Giulia Buzzatti, Chiara Molinelli, Piero Fregatti, Sergio Bertoglio, Francesco Boccardo, Lucia Del Mastro

**Affiliations:** 1 Department of Medical Oncology U.O. Oncologia Medica 2, IRCCS Ospedale Policlinico San Martino, Largo R. Benzi 10, 16132 Genova, Italy; 2 Department of Medical Oncology U.O.C. Clinica di Oncologia Medica, IRCCS Ospedale Policlinico San Martino, Largo R. Benzi 10, 16132 Genova, Italy; 3 Department of Internal Medicine and Medical Specialties (DiMI), University of Genova, Viale Benedetto XV 10, Genova, 16132 Italy; 4 Department of Oncology, Transplants and new Technologies U.O. Oncologia Medica I, Ospedale S. Chiara, Azienda Ospedaliera Universitaria Pisana, Via Roma 67, 56100 Pisa, Italy; 5 Medical Oncology, Ospedale San Paolo, Via Genova 30, 17100 Savona, Italy; 6 Department of Integrated Diagnostic Surgical Sciences, U.O. Clinica di chirurgia senologica, IRCCS Ospedale Policlinico San Martino, Largo R. Benzi 10, 16132 Genova, Italy; 7 Department of Surgical Sciences (DISC), University of Genova, Largo Rosanna Benzi 10, 16132 Genova, Italy; 8 General Surgery Unit, Department of Surgery, IRCCS Ospedale Policlinico San Martino, Largo Rosanna Benzi 10, 16132 Genova, Italy

**Keywords:** Breast cancer, Chemotherapy

## Abstract

**Background:**

Adjuvant chemotherapy is the standard of care in high-risk early breast cancer patients. Dose-dense should be the preferred schedule of administration. However, its long-term benefit is unknown.

**Methods:**

In the Italian multicentre Phase 3 randomised MIG-1 trial, node-positive and high-risk node- negative breast cancer patients were randomised to receive six cycles of adjuvant fluorouracil, epirubicin and cyclophosphamide regimen administered every 3 (FEC21) or 2 (FEC14) weeks. The primary endpoint was overall survival (OS), and the secondary endpoint was event-free survival (EFS).

**Results:**

From 1992 to 1997, 1214 patients were included. Median follow-up was 15.8 years. In all, 15-year OS was 71% and 68% in the FEC14 and FEC21 groups, respectively (HR = 0.89; *p* = 0.25). In all, 15-year EFS was 47% and 43% in the FEC14 and FEC21 groups, respectively (HR = 0.87; *p* = 0.18). In a pre-planned subgroup analysis, among patients with hormone receptor-negative tumours, 15-year OS was 70% and 65% in the FEC14 and FEC21 groups, respectively (HR = 0.73; 95% CI: 0.51–1.06); 15-year EFS was 58% and 43% in the FEC14 and FEC21 groups, respectively (HR = 0.70; 95% CI: 0.51–0.96).

**Conclusions:**

Updated results from the MIG-1 study are numerically in favour of dose-dense chemotherapy, and suggest a long-term benefit of this approach in high-risk early breast cancer patients.

## Introduction

High-risk early breast cancer patients are candidates to receive adjuvant chemotherapy. Anthracycline and taxane-based regimens are superior to other chemotherapy combinations.^1^ Several trials have tried to establish the best regimen and schedule of their administration.^2–8^ With the introduction of granulocyte colony-stimulating factors, it became possible to reduce the interval between chemotherapy cycles, thus allowing the use of dose-dense (DD) regimens.

Most of the trials that investigated DD chemotherapy compared different regimen in the DD and standard-interval group, leading to a difficult interpretation of their results. Nevertheless, five trials^9–13^ had clean comparison between DD and standard-interval groups in terms of administered dose, number of cycles, type of drug and total dose of chemotherapy. In a recently published patient-level meta-analysis, DD chemotherapy was superior to standard-interval regimens^14^ at a median follow-up of 7.4 years. Only two published trials included in the meta-analysis^11,12^ reported the results after a follow-up longer than 5 years, but none of the trials reported results beyond 10 years of median follow-up.

In the MIG-1 trial, started in 1992, early breast cancer patients were randomly assigned to receive six cycles of the 5-floruracil, epirubicin and cyclophosphamide (FEC) regimen given every 2 (FEC14) or every 3 (FEC21) weeks.^9^ Considering the significant risk of relapse up to 20 years after diagnosis in breast cancer patients, particularly in those with oestrogen receptor-positive tumours,^15^ we decided to update the MIG-1 study in order to report the long-term results, which can be used to better establish the real impact of DD chemotherapy.

## Patients and methods

### Study design and treatment regimens

Details of MIG-1 study design were previously reported.^9^ Briefly, MIG-1 was an Italian, open-label, multicentre Phase 3 randomised trial in high-risk early breast cancer patients. High-risk status was defined as lymph node-positive disease (no more than ten involved axillary lymph nodes) or lymph node-negative with one or more of the following features: age ≤35 years, negative status for oestrogen and progesterone receptors (defined as less than 10 fmol of receptor per milligram of protein or less than 10% positive cells by immunohistochemical analysis), tumour size larger than 2 cm, poor histologic grade and high proliferative tumours (determined by a [3 H]thymidine-labelling index or by an S-phase fraction obtained with flow cytometry). Patients were to have no clinical or radiologic evidence of distant metastases and an adequate bone marrow, hepatic and renal function. The study was conducted at 21 Italian centres after Ethics Committee approval; written informed consent was obtained from all patients before trial enrolment.

Eligible patients were randomly assigned to receive six cycles of FEC chemotherapy (5-fluorouracil at 600 mg/m^2^, epirubicin at 60 mg/m^2^ and cyclophosphamide at 600 mg/m^2^ intravenously) administered every 2 weeks with the support of filgrastim (FEC14) or every 3 weeks (FEC21).

Patients with oestrogen- and/or progesterone receptor-positive tumours received tamoxifen at 20 mg/day for 5 years. Postoperative regional radiotherapy limited to the remaining breast was given to patients who received breast-conserving surgery.

### Endpoints and statistical analysis

The primary study endpoint was overall survival (OS), as estimated from the date of randomisation to the date of last contact or death from any cause. Event-free survival (EFS), distant disease-free survival (DDFS) and toxicity were secondary endpoints. The EFS event was defined as local relapse, distant relapse, second primary cancer or death from any cause, whichever came first. DDFS was calculated from the time from randomisation until distant relapse. In this analysis, we reported OS, EFS, DDFS and incidence of secondary primary malignancies at a median follow-up of 15 years.

As previously reported,^9^ the study’s primary hypothesis was that a 50% increase in the dose intensity of FEC would be associated with a 20% relative reduction in the hazard of death. This reduction corresponds to a 5–6% absolute increase in 5-year survival, which was estimated to be between 65 and 70% in the standard-interval group. For a type I error of 5 and 80% power, 700 patients per group had to be enrolled over a 4-year period. The study was closed early due to the sharply declining accrual related to competitive trials, and the planned accrual was not reached. All analyses were conducted according to the intention-to-treat principles, in that all patients randomly assigned to a treatment arm were considered as belonging to the arm to which they had been assigned at randomisation. OS, EFS and DDFS survival rates were obtained from Kaplan–Meier analyses, and the primary comparison between the two study groups was performed with the log-rank test. Subgroup analysis of OS and EFS was conducted among patients with hormone receptor-negative and hormone receptor-positive disease. Moreover, we updated the previously published^16^ exploratory retrospective analysis on the efficacy of dose-dense chemotherapy according to Human Epidermal Growth Factor Receptor 2 (HER2) status. HER2 status was assessed centrally by immunohistochemical (IIC) analysis. Patients were considered HER2-positive only if IIC analysis was scored as 3+ . At the time of the study, FISH testing was not widely available. To evaluate the role of various prognostic factors and to test for heterogeneity in the effect of the experimental treatment in the subgroups of patients identified by the various prognostic factors, a series of Cox proportional hazard models were fitted to overall survival and event-free survival data. All tests were two-sided. IBM SPSS software was used in all statistical analyses.

## Results

From November 1992 to June 1997, 1214 patients were randomly assigned to receive FEC14 (*n* = 604) or FEC21 (*n* = 610) chemotherapy. Baseline characteristics at study entry were previously reported.^9^ Briefly, median age at study entry was 53 years. A total of 288 (48%) patients in the FEC14 group and 310 (51%) in the FEC21 group were diagnosed with tumour ≤ 2.0 cm, while 387 (64%) and 396 (65%) had positive lymph nodes, respectively (Table 1).

**Table 1:** Baseline characteristics by treatment group.

Characteristic	FEC14 (*N* = 604) *n* (%)	FEC21 (*N* = 610) *n* (%)
*Age*		
<50 y	250 (41)	220 (36)
≥50 y	354 (59)	390 (64)
*Menopausal status*		
Pre	265 (44)	259 (42)
Post	331 (55)	339 (56)
Unknown	8 (1)	12 (2)
*Tumour size, cm*		
≤2.0	288 (48)	310 (51)
2.1–5.0	285 (47)	257 (42)
≥5.1	25 (4)	35 (6)
Unknown	6 (1)	8 (1)
*Axillary lymph node status*		
Negative	217 (36)	214 (35)
pN1	260 (43)	243 (40)
pN2–3	124 (20)	151 (25)
Unknown	3 (0)	2 (0)
*Oestrogen receptor status*		
Negative	255 (42)	245 (40)
Positive	311 (51)	317 (52)
Unknown	38 (6)	48 (8)

*FEC* fluorouracil, epirubicin and cyclophosphamide, *FEC14* treatment administered every 14 days, *FEC21* treatment administered every 21 days, *pN1* 1–3 positive axillary lymph nodes, *pN2-3* ≥ 4 positive axillary lymph nodes.

**Fig. 1:**
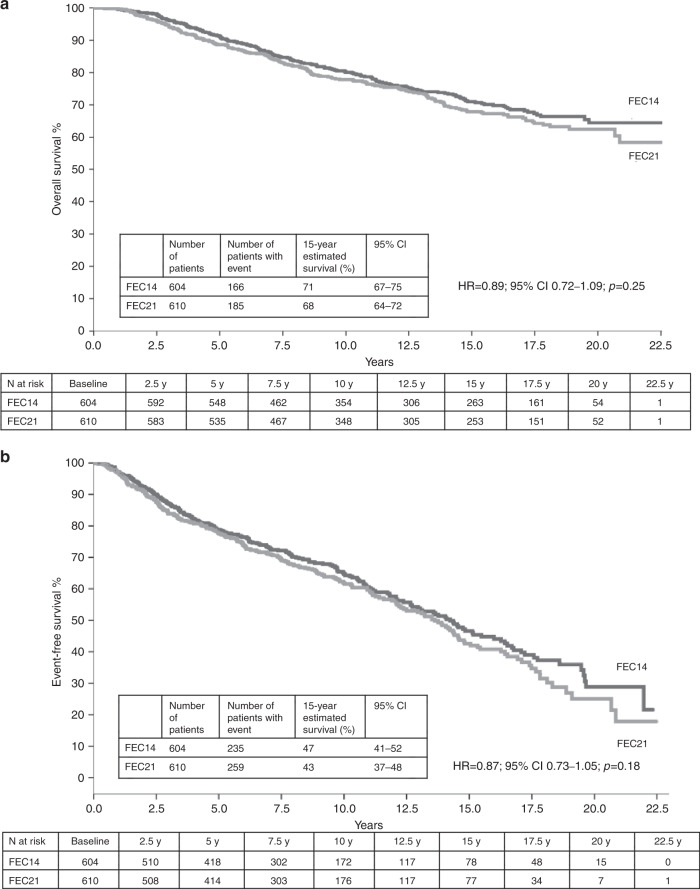
Kaplan–Meier survival curves for all randomly assigned patients. **a** Kaplan–Meier curve for OS among all patients. **b** Kaplan–Meier curve for EFS among all patients. FEC 5-fluorouracil, epirubicin and cyclophosphamide, FEC14 FEC administered every 2 weeks (dose-dense), FEC21 FEC administered every 3 weeks (standard interval).

At a median follow-up of 15.8 years, 351 deaths were observed: 166 (27%) in the FEC14 group and 185 (30%) in the FEC21 group. In all, 15-year OS was 71% (95% CI: 67–75) in the FEC14 group and 68% (95% CI: 64–72) in the FEC21 group (HR = 0.89; 95% CI: 0.72–1.09; *p* = 0.25) (Fig. 1a). In total, 494 events were observed: 235 (39%) in the FEC14 group and 259 (42%) in the FEC21 group. In all, 15-year EFS was 47% (95% CI: 41–52) in the FEC14 group and 43% (95% CI: 37–48) in the FEC21 group (HR = 0.87; 95% CI: 0.73–1.05; *p* = 0.18) (Fig. 1b). In all, 15-year DDFS rate was 72% (95% CI: 66–77) in the FEC14 group and 72% (95% CI: 68–77) in the FEC21 group (HR = 1.08; 95% CI: 0.84–1.39; *p* = 0.55). At the multivariable analyses (Table 2), after adjusting for age, menopausal status, tumour size, lymph node status, grading, proliferative activity and oestrogen and progesterone receptor status, no significant difference was observed in OS (HR = 0.89, 95% CI: 0.72–1.09; *p* = 0.26) or EFS (HR = 0.87, 95% CI: 0.73–1.05; *p* = 0.14) between FEC21 and FEC14 regimen.

**Table 2:** Multivariable analysis: association of prognostic factors with overall survival and event-free survival.

Variable	OS		EFS	
	HR (95% CI)	*P*	HR (95% CI)	*P*
*Random assignment*		0.25		0.18
FEC21	1 (ref.)		1 (ref.)	
FEC14	0.89 (0.72–1.09)		0.87 (0.73–1.05)	
*Tumour size*		<0.001		<0.001
≤2.0 cm	1 (ref.)		1 (ref.)	
>2.0 cm	1.89 (1.52–2.36)		1.62 (1.34–1.94)	
*Axillary lymph node status*		<0.001		<0.001
Negative	1 (ref.)		1 (ref.)	
Positive	2.22 (1.72–2.87)		2.08 (1.68–2.59)	
*Tumour grade*		0.06		0.35
G1	1 (ref.)		1 (ref.)	
G2	1.03 (0.61–1.73)		1.20 (0.79–1.82)	
G3	1.39 (0.82–2.37)		1.34 (0.87–2.07)	
Unknown	1.40 (0.78–2.52)		1.43 (0.89–2.30)	
*Menopausal status*		0.39		0.64
Pre	1 (ref.)		1 (ref.)	
Post	1.18 (0.81–1.74)		1.08 (0.79–1.46)	

*HR* hazard ratio, *CI* confidence interval, *ref.* referent, *FEC* fluorouracil, epirubicin and cyclophosphamide, *FEC14* treatment administered every 14 days, *FEC21* treatment administered every 21 days.

The incidence of second primary cancers was similar in the two groups, being 7.3% in the FEC14 group and 8.2% in the FEC21 group. Most of these events (4.4%) were ipsilateral or contralateral breast cancers; other second primary cancers were gastrointestinal (1.0%), gynaecological (1.0%), urological (0.4%), thyroid (0.4%), skin (0.2%), lung (0.2%), head and neck malignancies (0.2%) and myeloma (0.1%). No cases of acute leukaemia or myelodysplasia have been reported in patients in either arm.

### Subgroup analysis

In the subgroup analysis, we found no statistical evidence that the effect of the treatment type was significantly associated with age, tumour size, lymph node status, oestrogen and progesterone receptor status, proliferative rate, tumour grade or menopausal status (Table 3).

**Table 3:** Subgroup analysis of overall survival and event-free survival comparing FEC14 group with FEC21 group within strata formed by each prognostic factor.

Prognostic factor	HR (95% CI)	*P*	HR (95% CI)	*P*
*Age*		0.58		0.45
<50 y	0.99 (0.65–1.50)		0.82 (0.59–1.15)	
50–59 y	0.60 (0.40–0.91)		0.64 (0.45–0.91)	
>59 y	1.05 (0.72–1.55)		1.15 (0.82–1.63)	
*Menopausal status*		0.34		0.32
Pre	0.88 (0.59–1.30)		0.79 (0.58–1.08)	
Post	0.87 (0.66–1.16)		0.90 (0.70–1.16)	
*Tumour size*		0.24		0.80
≤2.0 cm	0.72 (0.49–1.06)		0.85 (0.63–1.16)	
Other	1.02 (0.76–1.36)		0.90 (0.69–1.16)	
*Axillary lymph node status*		0.87		0.48
Negative	0.89 (0.57–1.39)		0.73 (0.50–1.07)	
Positive	0.92 (0.71–1.20)		0.94 (0.75–1.18)	
*Tumour grade*		0.24		0.21
G1	1.27 (0.40–4.08)		0.60 (0.22–1.65)	
G2	0.78 (0.56–1.09)		0.85 (0.65–1.12)	
G3	0.93 (0.64–1.35)		0.82 (0.59–1.16)	
Unknown	1.91 (0.77–4.77)		2.86 (1.25–6.51)	
*Oestrogen receptor status*		0.34		0.15
Negative	0.73 (0.52–1.02)		0.70 (0.52–0.93)	
Positive	1.04 (0.76–1.41)		1.09 (0.84–1.42)	
*Progesterone receptor status*		0.98		0.55
Negative	0.85 (0.63–1.15)		0.80 (0.61–1.04)	
Positive	0.88 (0.62–1.27)		0.94 (0.69–1.26)	
*Proliferative rate*		0.29		0.17
Low	0.89 (0.51–1.54)		0.86 (0.54–1.36)	
High	0.68 (0.47–0.99)		0.72 (0.53–0.99)	
Unknown	1.12 (0.79–1.59)		1.10 (0.81–1.50)	

*HR* hazard ratio comparing FEC14 with FEC21, *CI* confidence interval, *FEC* fluorouracil, epirubicin and cyclophosphamide, *FEC14* treatment administered every 14 days, *FEC21* treatment administered every 21 days, *p*-value for interaction.

**Fig. 2:**
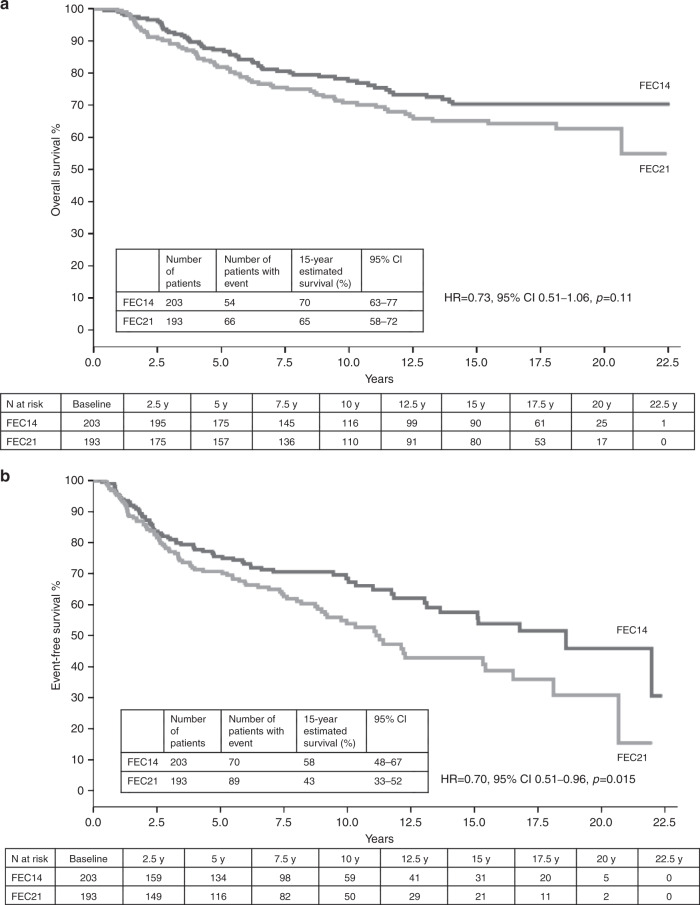
Kaplan–Meier curves for hormone receptor-negative patients. **a** OS. **b** EFS. FEC 5-fluorouracil, epirubicin and cyclophosphamide, FEC14 FEC administered every 2 weeks (dose-dense), FEC21 FEC administered every 3 weeks (standard interval).

Among patients with hormone receptor-negative tumours (*n* = 396), 15-year OS was 70% (95% CI: 63–77) in the FEC14 group and 65% (95% CI: 58–72) in the FEC21 group (HR = 0.73, 95% CI: 0.51–1.06, *p* = 0.11; *p*_interaction_ = 0.22) (Fig. 2a); 15-year EFS was 58% (95% CI: 48–67) in the FEC14 group and 43% (95% CI: 33–52) in the FEC21 group (HR = 0.70, 95% CI: 0.51–0.96, *p* = 0.015; *p*_interaction_ = 0.02) (Fig. 2b). In all, 15-year DDFS rate for hormone receptor-negative patients was 76% (95% CI: 68–83) in the FEC14 group and 70% (95% CI: 61–78) in the FEC21 group (HR = 0.79; 95% CI: 0.51–1.22; *p* = 0.28).

**Fig. 3:**
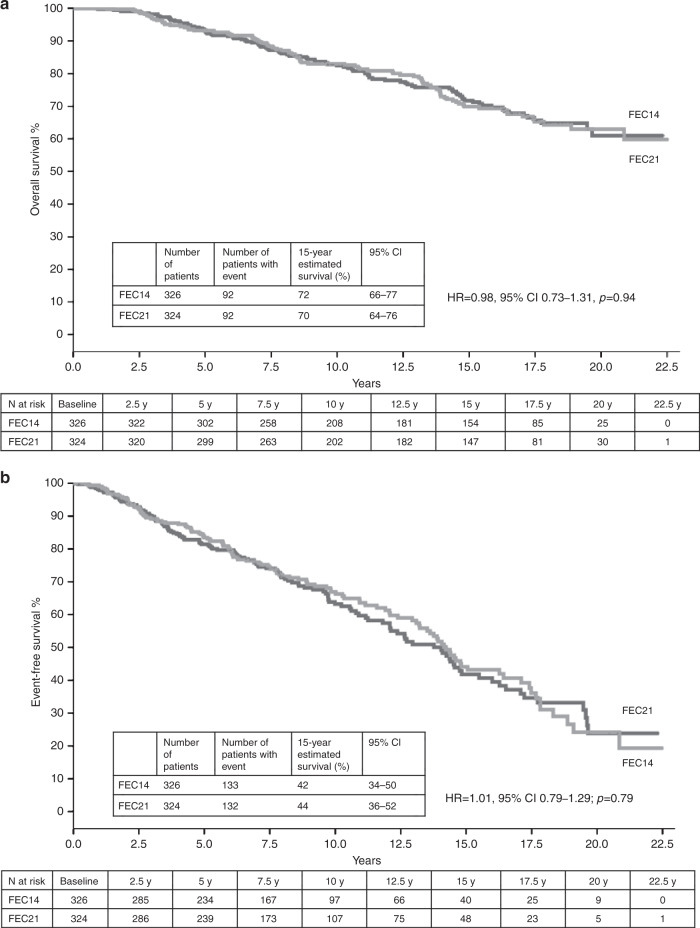
Kaplan–Meier curves for hormone receptor-positive patients. **a** OS. **b** EFS. FEC 5-fluorouracil, epirubicin and cyclophosphamide, FEC14 FEC administered every 2 weeks (dose-dense), FEC21 FEC administered every 3 weeks (standard interval).

Among patients with hormone receptor-positive tumours, 15-year OS was 72% (95% CI: 66–77) in the FEC14 group and 70% (95% CI: 64–76) in the FEC21 group (HR = 0.98, 95% CI: 0.73–1.31, *p* = 0.94) (Fig. 3a); 15-year EFS was 42% (95% CI: 34–50) in the FEC14 group and 44% (95% CI: 36–52) in the FEC21 group (HR = 1.01, 95% CI: 0.79–1.29; *p* = 0.79) (Fig. 3b). A 15-year DDFS rate for hormone receptor-positive patients was 70% (95% CI: 62–78) in the FEC14 group and 74% (95% CI: 68–81) in the FEC21 group (HR = 1.04; 95% CI: 0.73–1.47; *p* = 0.85).

Among the 731 patients with available data on HER2 status, 628 (86%) patients had a negative HER2 status, while 103 (14%) patients had a positive HER2 status. Among the patients with HER2-negative breast cancer, 15-year OS was 74% (95% CI: 68–79) in the FEC14 group and 70% (95% CI: 64–75) in the FEC21 group (HR = 0.82, 95% CI: 0.61–1.10, *p* = 0.19); 15-year EFS was 45% (95% CI: 37–53) in the FEC14 group and 44% (95% CI: 35–52) in the FEC21 group (HR = 0.88, 95% CI: 0.69–1.12; *p* = 0.30). While among the patients with HER2-positive breast cancer, 15-year OS was 70% (95% CI: 57–83) in the FEC14 group and 57% (95% CI: 43–72) in the FEC21 group (HR = 0.72, 95% CI: 0.39–1.35, *p* = 0.30); 15-year EFS was 57% (95% CI: 39–74) in the FEC14 group and 23% (95% CI: 7–39) in the FEC21 group (HR = 0.48, 95% CI: 0.28–0.84; *p* = 0.01).

## Discussion

We report long-term efficacy and safety outcomes of the MIG-1 trial that compared DD versus standard-interval FEC chemotherapy in high-risk early breast cancer patients. MIG-1 was a study of pure accelerated chemotherapy in that patients were randomised to FEC chemotherapy given at either 14- or 21-day intervals, with the same dose of drugs in both treatment arms.

With a median follow-up of 15.8 years, this is the study with the longest-term data reported so far. The reduction in the risk of death in the overall population (HR 0.89) is very close to the results observed in the individual patient-level meta-analysis, where, at a median follow-up of 7.4 years, a HR of 0.87 was reported.^14^ Likewise, the reduction in the risk of recurrence in our updated analysis (HR 0.87) is very similar with that reported in the meta-analysis (HR 0.86).^14^ The results of the analysis in most subgroups were numerically in favour of chemotherapy acceleration. The trend towards a greater benefit of DD  chemotherapy in hormone receptor-negative patients (HR for EFS 0.70, 95% CI: 0.51–0.96) and in HER2-positive patients (HR for EFS 0.48, 95% CI: 0.28–0.84) is hypothesis-generating. It can be hypothesised that DD chemotherapy works well in the subset of patients generally defined as sensitive to chemotherapy, such as hormone receptor-negative^17^ and HER2-positive patients.^18^ In this latter subgroup, a recent exploratory analysis suggests that the benefit of DD chemotherapy is lost when patients are treated with trastuzumab.^19^

Beside the long-term result of the DD approach, our analysis provides survival estimates for high-risk early breast cancer patients treated with anthracycline only-based chemotherapy. We observed an absolute 47% and 71% 15-year EFS and OS, respectively, for patients receiving DD chemotherapy. Notably, in patients with hormone receptor-positive tumours that received DD chemotherapy and endocrine therapy with tamoxifen for 5 years, 15-year EFS and OS were 42% and 72%, respectively. This poor long-term prognosis may suggest that anthracycline-only-based chemotherapy is an undertreatment in high-risk breast cancer patients, including those with hormone receptor-positive tumours.

In our trial, some limitations should be acknowledged. The study closed early and the planned accrual was not reached, so the statistical power is reduced. Due to the low accrual and the low mortality rate in the study population compared with the mortality expected by the original study plan, our study was underpowered to detect the planned risk reduction in the initial publication.^9^ With 351 deaths and 494 recorded events, our study had an 80% power to detect a reduction in the hazard of death of 27% and a reduction in the hazard of event of 20%, compared with the planned target difference of 20% reduction in the hazard of death. Moreover, an anthracycline-only chemotherapy regimen was used as adjuvant chemotherapy. This is no longer considered a standard of care in this setting, but it is still adopted by some centres for low-risk luminal-like breast cancer patients.^20,21^ Epirubicin was administered at a lower dose (60 mg/m^2^) than currently adopted; tamoxifen was the only adjuvant hormonal therapy administered to patients with hormone receptor-positive tumours. As well, trastuzumab and aromatase inhibitors were not available at the time of the trial. Postoperative adjuvant radiotherapy was administered only to patients undergoing breast-conserving surgery, whereas nowadays, post-mastectomy radiotherapy is administered to high-risk patients. No cardiac monitoring was planned during treatment or follow-up among patients enrolled in the study; thus, no data on cardiac safety are available.

In conclusion, our long-term results are similar to those reported in the individual-patient meta-analysis, and support that the benefit of DD chemotherapy is real and sustained to at least 15 years.

## Data Availability

Data and results are available at IRCCS Ospedale Policlinico San Martino, Genova, Italy.

## References

[1] Peto R, Davies C, Godwin J, Gray R, Pan HC, Early Breast Cancer Trialists’ Collaborative Group (EBCTCG (2012). Comparisons between different polychemotherapy regimens for early breast cancer: meta-analyses of long-term outcome among 100,000 women in 123 randomised trials. Lancet.

[2] Early Breast Cancer Trialists’ Collaborative Group (EBCTCG (2005). Effects of chemotherapy and hormonal therapy for early breast cancer on recurrence and 15-year survival: an overview of the randomised trials. Lancet.

[3] Seidman AD, Hudis CA, Albanell J, Tong W, Tepler I, Currie V (1998). Dose-dense therapy with weekly 1-hour paclitaxel infusions in the treatment of metastatic breast cancer. J. Clin. Oncol..

[4] Moebus V, Jackisch C, Lueck HJ, du Bois A, Thomssen C, Kurbacher C (2010). Intense dose-dense sequential chemotherapy with epirubicin, paclitaxel, and cyclophosphamide compared with conventionally scheduled chemotherapy in high-risk primary breast cancer: mature results of an AGO phase III study. J. Clin. Oncol..

[5] Therasse P, Mauriac L, Welnicka-Jaskiewicz M, Bruning P, Cufer T, Bonnefoi H (2003). Final results of a randomized phase III trial comparing cyclophosphamide, epirubicin, and fluorouracil with a dose-intensified epirubicin and cyclophosphamide + filgrastim as neoadjuvant treatment in locally advanced breast cancer: an EORTC-NCIC-SAKK multicenter study. J. Clin. Oncol..

[6] von Minckwitz G, Raab G, Caputo A, Schütte M, Hilfrich J, Blohmer JU (2005). Doxorubicin with cyclophosphamide followed by docetaxel every 21 days compared with doxorubicin and docetaxel every 14 days as preoperative treatment in operable breast cancer: the GEPARDUO study of the German Breast Group. J. Clin. Oncol..

[7] Kümmel S, Krocker J, Kohls A, Breitbach GP, Morack G, Budner M (2006). Randomised trial: survival benefit and safety of adjuvant dose-dense chemotherapy for node-positive breast cancer. Br. J. Cancer.

[8] Untch M, Möbus V, Kuhn W, Muck BR, Thomssen C, Bauerfeind I (2009). Intensive dose-dense compared with conventionally scheduled preoperative chemotherapy for high-risk primary breast cancer. J. Clin. Oncol..

[9] Venturini M, Del Mastro L, Aitini E, Baldini E, Caroti C, Contu A (2005). Dose-dense adjuvant chemotherapy in early breast cancer patients: results from a randomized trial. J. Natl Cancer Inst..

[10] Baldini E, Gardin G, Giannessi PG, Evangelista G, Roncella M, Prochilo T (2003). Accelerated versus standard cyclophosphamide, epirubicin and 5-fluorouracil or cyclophosphamide, methotrexate and 5-fluorouracil: a randomized phase III trial in locally advanced breast cancer. Ann. Oncol..

[11] Del Mastro L, De Placido S, Bruzzi P, De Laurentiis M, Boni C, Cavazzini G (2015). Fluorouracil and dose-dense chemotherapy in adjuvant treatment of patients with early-stage breast cancer: an open-label, 2 × 2 factorial, randomised phase 3 trial. Lancet.

[12] Cameron D, Morden JP, Canney P, Velikova G, Coleman R, Bartlett J (2017). Accelerated versus standard epirubicin followed by cyclophosphamide, methotrexate, and fluorouracil or capecitabine as adjuvant therapy for breast cancer in the randomised UK TACT2 trial (CRUK/05/19): a multicentre, phase 3, open-label, randomised, controlled trial. Lancet Oncol..

[13] Citron ML, Berry DA, Cirrincione C, Hudis C, Winer EP, Gradishar WJ (2003). Randomized trial of dose-dense versus conventionally scheduled and sequential versus concurrent combination chemotherapy as postoperative adjuvant treatment of node-positive primary breast cancer: first report of Intergroup Trial C9741/Cancer and Leukemia Group B Trial 9741. J. Clin. Oncol..

[14] Early Breast Cancer Trialists’ Collaborative Group (EBCTCG) (2019). Increasing the dose intensity of chemotherapy by more frequent administration or sequential scheduling: a patient-level meta-analysis of 37 298 women with early breast cancer in 26 randomised trials. Lancet.

[15] Pan H, Gray R, Braybrooke J, Davies C, Taylor C, McGale P (2017). 20-Year risks of breast-cancer recurrence after stopping endocrine therapy at 5 years. N. Engl. J. Med..

[16] Del Mastro L, Bruzzi P, Nicolò G, Cavazzini G, Contu A, D’Amico M (2005). HER2 expression and efficacy of dose-dense anthracycline-containing adjuvant chemotherapy in breast cancer patients. Br. J. Cancer.

[17] Colleoni M, Minchella I, Mazzarol G, Nolè F, Peruzzotti G, Rocca A (2000). Response to primary chemotherapy in breast cancer patients with tumors not expressing estrogen and progesterone receptors. Ann. Oncol..

[18] Borg A, Baldetorp B, Fernö M, Killander D, Olsson H, Sigurdsson H (1991). ERBB2 amplification in breast cancer with a high rate of proliferation. Oncogene.

[19] Lambertini Matteo, Poggio Francesca, Bruzzone Marco, Conte Benedetta, Bighin Claudia, Azambuja Evandro, Giuliano Mario, De Laurentiis Michele, Cognetti Francesco, Fabi Alessandra, Bisagni Giancarlo, Durando Antonio, Turletti Anna, Urracci Ylenia, Garrone Ornella, Puglisi Fabio, Montemurro Filippo, Ceppi Marcello, Del Mastro Lucia (2019). Dose‐dense adjuvant chemotherapy in HER2‐positive early breast cancer patients before and after the introduction of trastuzumab: Exploratory analysis of the GIM2 trial. International Journal of Cancer.

[20] Curigliano G, Criscitiello C, Esposito A, Pruneri G (2017). Over-using chemotherapy in the adjuvant setting. Breast.

[21] Munzone E, Curigliano G, Colleoni M (2013). Tailoring adjuvant treatments for the individual patient with luminal breast cancer. Hematol. Oncol. Clin. North Am..

